# Imperforate Anus—the Rectum Communicating with the Vagina

**Published:** 1879-11

**Authors:** Thos. Lothrop


### IMPERFORATE ANUS.

### THE RECTUM COMMUNICATING WITH THE VAGINA.

REPORTED BY THOS. LOTHROP, M. D.

In an able paper, read before the New York Obstetrical Society, in January, 1872, Dr. J. H. Pooley reported a case of this malformation, which is the rarest variety of imperforate anus. In the same valuable contribution is a record of thirty-seven cases, all that could be found, some of which date back to the early part of the sixteenth century. The extreme rarity of the malformation, and the interest existing in the profession to preserve a record of all cases, especially of congenital defects, is my apology for this "clinical record."

Mrs. F., aged 35 years, German, was delivered May 5, 1874, of a female child. The labor was a natural one, the mother and child requiring only the care and attention given by the accoucheur in ordinary cases. The usual inquiries were made the following day in regard to the child, its power of evacuating fæces and urine, to which satisfactory answers were given by the attendants. As soon as the mother had recovered sufficiently to care for her child, observing that there was something unusual in its manner of evacuating the bowels, she summoned me. Examination revealed an imperforate anus, and in passing a small probe into the vagina, an opening in the recto-vaginal septum was observed, just within the os vaginæ the orifice being easy of detection, the fæcal matter passing at the time of the examination. The natural site of the anus was marked by a discolored spot, with lines or plicae of the integument radiating from it. The advisability of an operation was laid before the parents, who objected to any interference, on being assured that there was no immediate danger to the life of the child. Frequent importunities to open the anus failed to change the decision of the mother, and operative interference was not resorted to. In the following August, the child was attacked with gastrointestinal disease, to which it rapidly succumbed, its death occurring at the age of four months.

This malformation possesses many features of professional interest. It is not necessarily dangerous to life; one of the reported cases, it is stated, lived one hundred years; three are stated to have borne children; and others failed to discover the deformity until they reached adult life; others reached the ages respectively of twenty, twenty-two, seventeen and sixteen years. The fistulous opening in these exceptional cases must have been very large, and therefore sufficient to answer the demands of nature. Accumulations may occur, the fæcal evacuations being insufficient to unload the intestines, as in a case reported of a child who lived to the age of four and one-half years, the colon being distended to such an extent as to encroach on the thorax. It is therefore the duty of the surgeon to advise the operation whenever he is consulted in these cases, even in the first month or two of life. It consists of two parts, first to secure an opening at the normal site of the anus, and second to close up the fistulous orifice. Two successful operations are reported, one by the late Dr. Parrish, and the second by Dr. Rhea Barton, formerly surgeon of the Pennsylvania Hospital. The following is the mode of operation adopted: Introduce a director into the fistulous opening, and with a bistoury lay open the vagina and integuments as far back as the part where the anus should be; then remove a small portion of the integuments if necessary, and dissect down, until the termination of the gut is reached, which is opened freely. By this method, the anterior boundary of the incision would be the fistulous opening in the vagina, and posteriorly, it would terminate where the natural outlet ought to be. The subsequent treatment consists in endeavoring to promote granulations and the cicatrizing of the original opening, and so much of the anterior portion of incision as rendered the vagina incomplete. Both operations were successful and the patients were able to retain or discharge fæces at pleasure.

